# Genome-Wide Modulation of Gene Transcription in Ovarian Carcinoma Cells by a New Mithramycin Analogue

**DOI:** 10.1371/journal.pone.0104687

**Published:** 2014-08-11

**Authors:** Carolina Vizcaíno, Luz-Elena Núñez, Francisco Morís, José Portugal

**Affiliations:** 1 Instituto de Biología Molecular de Barcelona, Consejo Superior de Investigaciones Cientificas, Barcelona, Spain; 2 EntreChem, Oviedo, Spain; Università degli Studi di Milano, Italy

## Abstract

Ovarian cancer has a poor prognosis due to intrinsic or acquired resistance to some cytotoxic drugs, raising the interest in new DNA-binding agents such as mithramycin analogues as potential chemotherapeutic agents in gynecological cancer. Using a genome-wide approach, we have analyzed gene expression in A2780 human ovarian carcinoma cells treated with the novel mithramycin analogue DIG-MSK (demycarosyl-3D-β-D-digitoxosyl-mithramycin SK) that binds to C+G-rich DNA sequences. Nanomolar concentrations of DIG-MSK abrogated the expression of genes involved in a variety of cell processes including transcription regulation and tumor development, which resulted in cell death. Some of those genes have been associated with cell proliferation and poor prognosis in ovarian cancer. Sp1 transcription factor regulated most of the genes that were down-regulated by the drug, as well as the up-regulation of other genes mainly involved in response to cell stress. The effect of DIG-MSK in the control of gene expression by other transcription factors was also explored. Some of them, such as CREB, E2F and EGR1, also recognize C/G-rich regions in gene promoters, which encompass potential DIG-MSK binding sites. DIG-MSK affected several biological processes and molecular functions related to transcription and its cellular regulation in A2780 cells, including transcription factor activity. This new compound might be a promising drug for the treatment of ovarian cancer.

## Introduction

Ovarian cancer is an important cause of morbidity and mortality worldwide and the main cause of death among gynecological cancers [1], [2]. Surgery followed by platinum-taxane chemotherapy is the standard treatment for ovarian cancer [3]. Despite improvements in complete clinical remission and progression-free survival, resistance to chemotherapy presents a major problem in the treatment of ovarian cancer and a contributing factor in cancer-associated mortality [1]–[3]. Therefore, given that ovarian cancer shows a high risk of relapse, it seems necessary to improve the efficacy of novel targeted therapies [4].

Regulation of gene transcription is often a central point in oncogenic signaling [5]. In ovarian cancer, an integrated genomic analysis has been undertaken [6], and there have been intents to assess the association between transcription, overall survival and response to chemotherapy [7], [8]. In this context, identifying transcription factors that are involved in tumorigenesis and cancer progression may provide us with targets for chemotherapeutic intervention based on small compounds [5], [9]. Although targeting transcription factors and their interactions with gene promoters is a difficult approach, it is nowadays considered an attainable goal [5], [9]. In fact, many clinically useful agents, such as the anthracyclines doxorubicin and daunorubicin, several alkylating agents and mithramycin A, can regulate gene expression by binding to C+G-rich DNA sequences recognized by the Sp-family of transcription factors, thus abrogating the transcriptional activity of genes essential for cancer cell growth [9], [10]. Ovarian cancer cells over-express several genes that contribute to tumor development [6], [11]–[15]. In many cases, these genes are activated by Sp1 [16]–[18] and/or by other transcription factors [7], representing potential targets for therapeutic intervention.

Mithramycin A (MTA) is an aureolic acid-type polyketide antibiotic produced by various species of *Streptomyces* [19]. It has been used in the treatment of Paget’s disease and advanced testicular carcinoma, but it showed numerous toxic side effects which have limited its clinical use [20]. At the molecular level, the activity of MTA and several of its analogues has been associated with their ability to bind to C/G-rich regions within the DNA minor groove [21]–[23], and references therein. At present, MTA is the subject of ongoing clinical trials in Ewing sarcoma (http://www.ClinicalTrials.gov; Identifier: NCT01610570) and lung, esophagus and other chest cancers (http://www.ClinicalTrials.gov; Identifier: NCT01624090), indicating a renewed interest in this kind of compounds for clinical cancer treatment.

**Figure 1:**
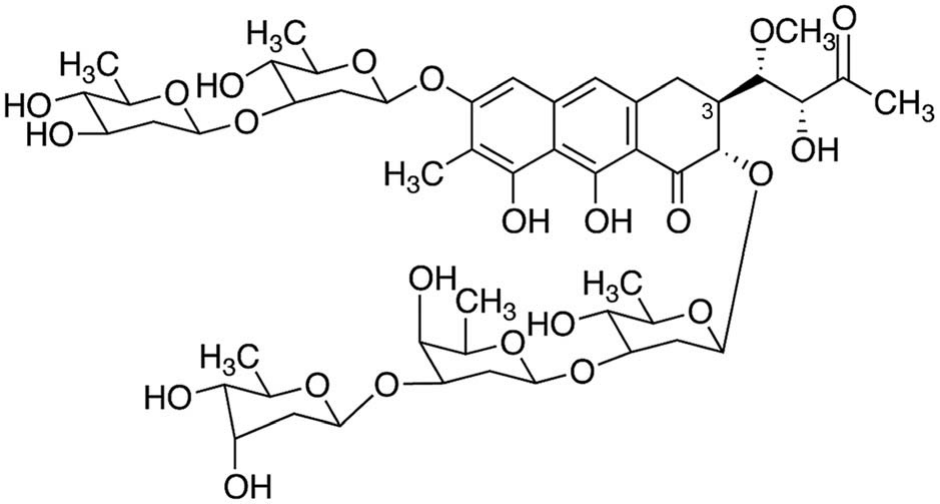
Chemical formulae of the mithramycin analogue DIG-MSK (demycarosyl-3D-β-D-digitoxosyl-mithramycin SK). DIG-MSK differs from the parental mithramycin A in the side chain linked to C-3 and the distal sugar in the trisaccharide moiety.

The genetic organization of the MTA biosynthesis gene cluster has been studied in detail, and the MTA biosynthetic pathway used to produce new compounds with enhanced biological characteristics [19], [21], [24]–[26]. New mithramycin analogues bearing both lower toxicity and higher biological activity are now available, providing new possibilities for therapeutic application [18], [19], [25]. MTA and its analogues tested to date can inhibit transcription both *in vivo* and *in vitro* by interfering with protein-DNA interactions, especially the inhibition of Sp1-dependent transcription [17], [18], [27]–[29]. Recently, a new analogue named DIG-MSK (demycarosyl-3D-β-D-digitoxosyl-mithramycin SK; EC-8042) (Fig. 1) has been obtained and characterized [25]. DIG-MSK shows *in vivo* and *in vitro* antitumor activities similar to other novel analogues like the structurally related MSK, but DIG-MSK is 10-fold less toxic *in vivo* than MTA and 25% less toxic than MSK [25]. Remarkably, the single maximum tolerated dose of DIG-MSK in mice is the highest among the mithramycin analogues [25]. DIG-MSK inhibits the growth of HCT-116 human colon carcinoma cells, where it inhibits the interaction between transcription factors and DNA [29]. Moreover, the *in vivo* evaluation of DIG-MSK antitumor activity by hollow fiber assays indicates that it is a promising antitumor drug against ovarian cancer, among other neoplasms [25].

Using A2780 human ovarian carcinoma cells, we sought to characterize the effects of the mithramycin analogue DIG-MSK on transcription through a genome-wide analysis of changes in gene expression. As expected, we observed this compound reduced the expression of a variety of genes, many of which have been related with ovarian cancer progression, but also up-regulates the expression of other genes, consistent with the stress response that chemotherapeutic drugs can produce in treated cells. We postulate that the effects of DIG-MSK on gene transcription are mainly due to interference with the binding of Sp1 to its putative binding sites in gene promoters, yet we also observed that other transcription factors were modulated by the drug in their binding to gene promoters, in keeping with our previous observations in colon carcinoma cells [29]. Besides, several biological processes and molecular functions related to transcription and its cellular regulation, including transcription factor activity, were significantly influenced by DIG-MSK in A2780 cells.

## Materials and Methods

### Cell culture and drug treatments

A2780 human ovarian carcinoma cells were growth in RPMI 1640 medium (Life Technologies) supplemented with 10% fetal bovine serum (Life Technologies), 100 U/ml penicillin, 100 µg/ml streptomycin and 2 mM sodium pyruvate at 37°C in a humidified atmosphere with 5% CO_2_.

Demycarosil-3D-β-D-digitoxosyl mithramycin SK (DIG-MSK; EC-8042) (Fig. 1) was isolated and purified (HPLC≥97%) from the producer *Streptomyces* species as described elsewhere [25]. Stocks of DIG-MSK were prepared as 1 mM solutions in sterile 150 mM NaCl, maintained at −20°C, and brought to the final concentrations just before use. Exponentially growing cells subcultured at a density of 2.5×10^4^ cells/ml were grown in the presence of several concentrations of DIG-MSK in the nanomolar range.

### Assessment of cell proliferation and viable cell number

The capacity of DIG-MSK to interfere with the growth of A2780 cells proliferation was determined by the MTT assay using 3-(4,5-dimethylthiazol-2-yl)-2,5-diphenyltetrazolium (Sigma, St. Louis, MO) as described elsewhere [30]. Briefly, cells subcultured at a density of 2.5×10^4^ cells/ml were incubated with a nanomolar range of drug concentrations at 37°C for 72 h, and MTT was added to each culture. Viable cell number was determined at different intervals based on the exclusion of Trypan blue dye and a hemocytometer.

### RNA preparation

Cell lysates were prepared from A2780 human ovarian carcinoma cells treated in triplicate with 8 nM or 80 nM DIG-MSK –see Results about how these drug concentrations were selected–, and from untreated cells. RNA was isolated from those independent biological replicates by using the Ultraspec RNA reagent (Biotecx) following the manufacturer’s recommendations. RNA was digested with RNAse-free DNAse I (Roche Diagnostics) in the presence of RNasin (Promega), phenol extracted and precipitated, and the pellets were dissolved in RNAse-free water. RNA was quantified using a NanoDrop spectrophotometer, and the integrity of total RNA monitored with an Agilent 2100 Bioanalyzer. All samples showed a RIN higher than 7.

### Microarray analysis

Total RNAs (0.2 µg) from each sample (biological replicates) were amplified, labeled and hybridized to a SurePrint G3 Human Gene Expression Microarray (Agilent, ID 028004). RNA labeling was performed with the Low Input Quick Amp Labeling kit (Agilent) following the manufacturer’s protocol. Oligoarray hybridization buffer (In Situ Hybridization Kit Plus (Agilent)) was added afterwards, and samples applied to microarrays enclosed in Agilent SureHyb-enabled hybridization chambers. Images of the microarrays were acquired using a G2505C Scanner (Agilent). Background subtraction and locally weighted scatterplot smoothing (lowess) normalization were performed using the Feature Extraction Software v. 10.7.3.1 (Agilent). Arrays were processed at Bioarray SL (Elche, Spain). All statistical and differential expression analyses were carried out with an empirical Bayes approach on linear models using the Limma package from Bioconductor (http://www.bioconductor.org/). Gene expression was considered up- or down-regulated when it changed ≥1.5-fold (up-regulated) or ≤0.67-fold (down-regulated) and statistically significant (*p*<0.05). The microarray data can be accessed at the Gene Expression Omnibus (GEO) database: http://www.ncbi.nlm.nih.gov/geo/query/acc.cgi?acc=GSE46926.

### Validating microarray data by qRT-PCR

An aliquot of the RNA preparations from untreated and drug-treated samples used in the microarray analysis were saved for validation by qRT-PCR. RNAs from each experimental condition were pulled together and analyzed in three independent quantifications. First strand cDNAs were synthesized from DNAse I-treated total RNA. The qRT-PCR reactions were performed in triplicate in a Roche LightCycler 480, using the SYBR-Green PCR Master Mix (Roche Diagnostics), and the primers shown in Table S1. PCR conditions included an initial denaturation step at 95°C for 10 min, followed by 45 cycles of a denaturation step at 95°C for 15 s and an annealing/extension step at 60°C for 1 min. A final dissociation curve was generated to verify that a single product was amplified. Reactions in the absence of template and in the absence of enzyme were also included as negative controls. Fold changes between treatments were determined by the ΔCt method [31], using the expression of the housekeeping *GAPDH* gene for data normalization.

### Gene Ontology (GO) analysis

Functional classification of the genes down-regulated upon treatment with DIG-MSK (≤0.67-fold change) in *Biological Process* and *Molecular Function* categories was performed by comparison with the list of all the genes studied by using PANTHER v.7 (http://www.pantherdb.org/tools/), which uses the binomial test for assessing statistical significance [32].

### Identification of transcription factors associated to genes affected by DIG-MSK

The Transcription Element Listening System (TELiS) [33] was used to analyze the prevalence of transcription factor-binding motifs, among the promoters of genes down-regulated upon exposure to DIG-MSK. The *Incidence analyses,* provided by the software, indicated genes bearing at least one binding site for a particular transcription factor that were present in a greater fraction of differentially expressed genes than in the sampling frame as a whole. This binary analysis was executed online as an exact binomial test at http://www.telis.ucla.edu/index.php?cmd=transfac; using default parameters (promoter size “−600” and stringency setting “0.9”). Consensus sequences corresponding to transcription-factor binding sites were located in the promoter regions of the genes down-regulated by DIG-MSK through multiple comparative analysis, and retrieved using TELiS.

Consensus transcription factor binding sites were identified using both the JASPAR database (http://jaspar.genereg.net/cgi-bin/jaspar_db.pl) and MatInspector 8.0 (Genomatix Software Suite).

An enrichment analysis was performed using Enrichr [34] to obtain an unbiased identification of whether genes down-regulated by DIG-MSK were among those described to be up-regulated in A2780 human carcinoma cells, thus they participate in the characteristics of A2780 transformed state. A list consisting of down-regulated genes (≤0.67-fold, *p*<0.05) was uploaded at: http://amp.pharm.mssm.edu/Enrichr, where it was compared with the Cancer Cell Line Encyclopedia [35]. The Fisher’s exact test, provided by Enrichr [34], was used to assess the statistical significance of the overlap between the input list and genes overexpressed in A2780 cells.

### Hierarchical clustering of selected data

Logarithmic (log_2_) values of the normalized expression ratios (treated *versus* untreated cells) were hierarchically clustered using the TIGR-MeV program [36], based on the Pearson correlations. A comparison was undertaken with the total of genes differentially expressed by 8 and 80 nM DIG-MSK for bibliographic co-citations, pathways and diseases (MeSH terms) in the Genomatix Pathways System (GePS) platform included in the Genomatix Software Suite (http://www.genomatix.de/solutions/genomatix-software-suite.html). A set of 32 genes was selected from the genes modulated by both 8 nM and 80 nM DIG-MSK in A2780 cells, filtered by *Diseases (MeSH) and Ovarian neoplasm* by using GePS in the Genomatix Software Suite, and for the presence of consensus Sp1 binding sites in their promoters using TELiS [33]. Details on the whole procedure are provided in Results.

#### Cell cycle analysis by flow cytometry

A2780 cells treated with DIG-MSK and untreated, control, cells were harvested at different periods of time, fixed with 70% ethanol, and stained with PI (propidium iodide, Sigma) as described elsewhere [30]. Nuclei were analyzed with a Coulter Epics-XL flow cytometer (Coulter Corporation), using the 488 nm line of an argon laser and standard optical emission filters.

### Assessment of the mechanisms of cell death

Apoptosis was quantified and distinguished from necrosis by using the Annexin-V-Fluos staining kit (Roche Diagnostics) and flow cytometry in a Coulter Epics-XL flow cytometer (Beckman Coulter).

### Western analysis

Protein was extracted from A2780 cells treated with 80 nM DIG-MSK and from untreated cells. About 20 µg of denatured proteins were subjected to electrophoresis on SDS-polyacrylamide gels, blotted onto Optitran BA-S85 membranes (Schleicher & Schuell), probed with specific antibodies: p21^WAF1^ (Calbiochem,), p53 (Santa Cruz), Sp1 (Santa Cruz), Sp3 (Santa Cruz), and GAPDH (Sigma), incubated with secondary antibodies (Jackson ImmunoResearch), and detected by chemiluminescence using the Immobilon Western HRP Substrate (Millipore).

### Chromatin immunoprecipitation (ChIP)

For chromatin immunoprecipitation (ChIP), about 10^7^ A2780 cells either untreated or treated with 80 nM DIG-MSK for 24 h were cross-linked with 1% formaldehyde for 10 min. After quenching formaldehyde, cells were collected, lysed, and washed with cold PBS. Chromatin was re-suspended in 4 ml of a buffer consisting of 1 mM EDTA, 0.1% SDS, 10 mM Tris-HCl (pH 7.4), containing protease inhibitors, and sonicated in a Bioruptor (Diagenode) for 25 min to obtain 200–500 bp fragments. Cross-linked chromatin was pre-cleared with 40 µl Dynabeads-Protein A (Invitrogen) and then immunoprecipitated with anti-Sp1 antibody (H-225X, Santa Cruz) in RIPA buffer (140 mM NaCl, 1 mM EDTA, 1% Triton X-100, 0.1% SDS, 0.1% sodium deoxycholate, 10 mM Tris-HCl (pH 8.0), and protease inhibitors) using 30 µl of pre-blocked Dynabeads-Protein A (Invitrogen). As a negative control, mock immunoprecipitations were carried out using rabbit IgG (Thermo Scientific). Subsequently, cross-links were reversed and DNA purified. qRT-PCR was performed on ChIP material using the primers for *XIAP, CRABP1, MDK, KCNMA,* and a *GAPDH* coding region lacking Sp1 binding sites –negative control– shown in Table S1, and the experimental conditions described above.

## Results

### DIG-MSK inhibits the proliferation of A2780 human ovarian carcinoma cells

**Figure 2:**
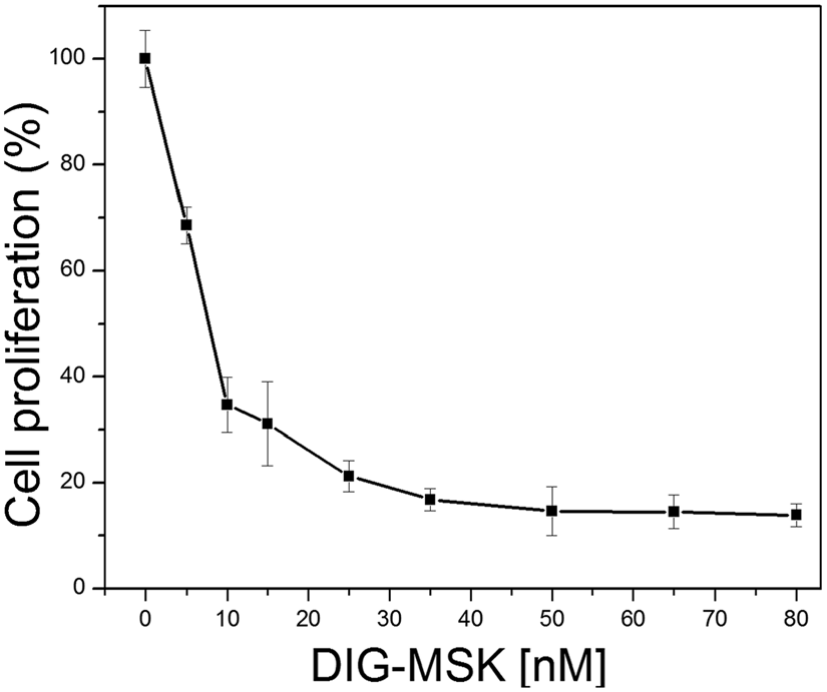
Assessment of cell proliferation. Effects of DIG-MSK on the proliferation of A2780 human ovarian carcinoma cells after 72-h continuous treatments. Data are mean ± SEM from 6 independent experiments.

Antiproliferative effects of nanomolar concentrations of DIG-MSK on A2780 cells were evaluated after 72-h continuous treatments (Fig. 2). From the plot in Fig. 2, we calculated the IC_50_ (drug concentration required to inhibit cell growth by 50%) and IC_75_ values (drug concentration required to inhibit cell growth by 75%) to be 4.25±0.39 nM and 7.44 ±0.12 nM, respectively. Moreover, the assessment of the reduction in viable cell number indicated that 8 nM DIG-MSK (∼its IC_75_ at 72 h) produced around 4% cell death at 24 h, which reached 9% in 72-h treatments. Besides, 80 nM DIG-MSK (i.e. 10-fold the IC_75_ concentration measured after 72-h treatments) produced ∼16% death after 24 h and 90% after 72-h treatments.

### DIG-MSK modulates gene expression in A2780 cells

**Figure 3:**
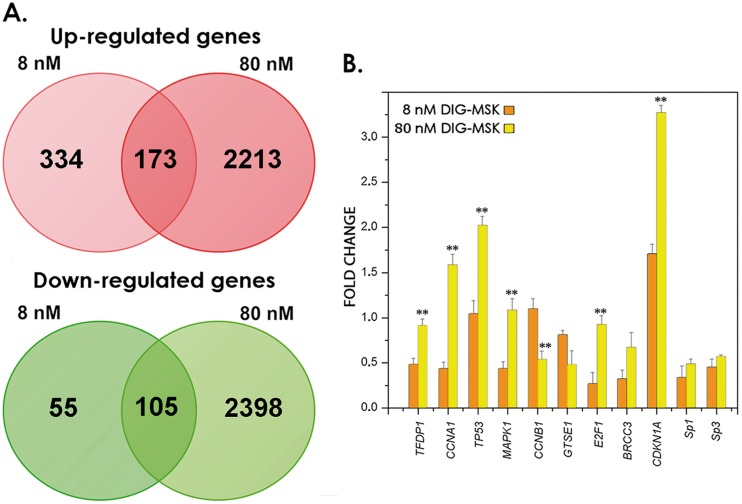
Analysis of gene expression in A2780 cells treated with DIG-MSK. (A) Venn diagrams representing genes affected, either up-regulated or down-regulated in microarray analysis by treatments with 8 nM or 80 nM DIG-MSK (1.5-fold changes, *p*<0.05). Numbers inside the intersections correspond to genes influenced by both drug concentrations. (B) Quantitative real-time PCR measurements of a set of genes selected among those differentially expressed in A2780 cells treated with DIG-MSK. Compared to untreated cells, the expression of all these genes changed significantly upon treatment (*p*<0.05). Histograms represent mean ± SD from 3 independent experiments (**p<0.01, Student’s t-test comparison between treatments with either 8 nM or 80 nM DIG-MSK).

We analyzed the effects of 8 and 80 nM DIG-MSK on gene expression in A2780 human ovarian cancer cells after 24-h treatments. These sub-lethal concentrations were selected from the analysis of cell proliferation (Fig. 2) and viability by Trypan blue exclusion by living cells. The supporting microarray data have been submitted to the GEO repository (http://www.ncbi.nlm.nih.gov/geo/query/acc.cgi?acc=GSE46926). After 24-h continuous treatments, 8 nM DIG-MSK affected the expression of 667 genes (1.5-fold; *p*<0.05), of which 160 were down-regulated (Fig. 3A). At the same threshold, 80 nM DIG-MSK affected 4889 transcripts, of which 2503 were down-regulated. Most of the genes showing altered expression were down-regulated by the higher concentration. Besides, 105 genes showed down-regulated gene expression at both concentrations (intersection in the Venn diagrams in Fig. 3A).

We sought independent validation by qRT-PCR of the DIG-MSK effects on gene expression. We selected 11 transcripts, that have been reported to be challenged by DIG-MSK in a different cancer cell type [29]. The genes analyzed by qRT-PCR are also related to ovarian cancer [6] and/or contain putative Sp1-binding sites. We observed substantial changes in gene expression upon treatment with DIG-MSK (Fig. 3B), in keeping with the microarray data, although for some transcripts the changes were less pronounced. Compared to control, untreated, cells those changes were statistically significant (p<0.05). Moreover, for several genes, differences between the effects of 8 nM and 80 nM DIG-MSK (Fig. 3B) were highly significant (p<0.01).

### Gene Ontology (GO) of genes differentially expressed upon treatments with DIG-MSK

Table 1 classifies down-regulated genes in gene ontology (GO) classes. This table also provides a statistical estimate of the overrepresentation of a given functional class. DIG-MSK affected a variety of biological routes, including genes related to transcription regulation and metabolic cellular processes. *Molecular function* and *biological processes* GO categories highlighted the presence of genes involved in transcription regulation and nucleic acids metabolism among the genes affected by 80 nM DIG-MSK, which suggested that DIG-MSK alters the expression, or binding activities, of a variety of transcriptional factors. A similar analysis was undertaken using the results of the treatment with 8 nM DIG-MSK (Table 2). In this case, both the number of genes and the GO categories represented significantly (*p*<0.05) were smaller, indicating a dose-dependent response to the drug.

**Table 1:** Gene Ontology (GO) categories affected by 80 nM DIG-MSK.

GO categories^a^	AgilentMicroarray(Ref. list)	Number ofdown- regulatedgenes	Number ofexpectedgenes	*p*-value^b^
***Biological process***				
Nucleobase, nucleoside, nucleotideand nucleic acid metabolic process	3101	571	385.44	4.69E-21
Transcription	1879	381	233.55	3.02E-19
Transcription from RNA polymeraseII promoter	1874	380	232.93	3.44E-19
Regulation of transcription from RNApolymerase II promoter	1493	306	185.57	1.54E-15
Primary metabolic process	6704	978	833.27	2.18E-08
Metabolic process	7060	1010	877.51	7.65E-07
System development	1167	212	145.05	6.27E-06
Establishment or maintenanceof chromatin architecture	228	58	28.34	9.58E-05
Organelle organization	253	60	31.45	5.42E-04
Developmental process	2022	317	251.32	1.96E-03
Cellular process	5081	713	631.54	1.29E-02
***Molecular function***				
Binding	5511	915	684.98	2.71E-23
DNA binding	2041	417	253.68	4.42E-22
Nucleic acid binding	3196	581	397.24	2.60E-20
Transcription factor activity	1817	364	225.84	1.77E-17
Transcription regulator activity	1817	364	225.84	1.77E-17
Transcription cofactor activity	162	44	20.14	3.74E-04
Structural constituent of ribosome	176	5	21.88	2.34E-03

a The GO analysis was undertaken on down-regulated genes by PANTHER 7.0.

b Binomial test.

**Table 2:** Gene Ontology (GO) categories affected by 8 nM DIG-MSK.

GO categories^a^	AgilentMicroarray(Ref. list)	Number ofDown- regulatedgenes	Number ofexpected genes	*p*-value^b^
***Biological process***				
Nucleobase, nucleoside,nucleotide and nucleic acidmetabolic process	3101	48	25.03	4.33E-4
Cell cycle	10.85	22	8.76	1.06E-02
Primary metabolic process	6704	74	54.11	7.69E-02
***Molecular function***				
Binding	5511	75	44.48	1.17E-05
DNA binding	3196	50	25.79	1.49E-04

a The GO analysis was undertaken on down-regulated genes by PANTHER 7.0.

b Binomial test.

### Identification of transcription factors associated with genes down-regulated by DIG-MSK

Given that the mithramycin analogues can be *in vivo* and *in vitro* inhibitors of gene transcription through direct competition with proteins for binding to DNA, we explored whether among transcription factors that recognize gene promoters there was an ‘enrichment’ in their putative binding sites in the promoters of down-regulated genes. This might explain the repression of gene expression by DIG-MSK. Drug-repressed genes encompassed in their promoters a higher proportion of binding sites for several transcription factors than it was expected to occur by simple chance (Table 3). Sp1 had the highest representation among the transcription factors, in line with DIG-MSK effects on Sp1-activated gene expression in human colon carcinoma cells [29], and consistent with the fact that Sp1 and the mithramycin analogues bind preferentially to C/G-rich regions in DNA. Other transcription factors that bind to G+C sequences and participate in the control of gene expression during cancer development, such as EGR2, N-Myc, or E2F, were among the more represented factors (Table 3).

**Table 3:** Transcription factors whose consensus binding sequences are over-represented in the promoter region of genes down-regulated by either 8 nM or 80 nM DIG-MSK in A2780 human ovarian carcinoma cells.

Transcriptionfactor		8 nM		80 nM	
	Consensus sequence^a^	Observed genesAG: 81^b^	Incidence	Observed genesAG: 1262^b^	Incidence
			*p*-value^c^		*p*-value^c^
**CREB**	GGTGACGTAAGG	19	1.0E-03	190	1.0E-10
**EGR1**	ATGCGTGGGCGT	//	//	20	2.2E-03
**EGR2**	A/TTGCGTGGGCGT	//	//	31	1.0E-10
**EGR3**	ATGCGTGGGCGT	3	4.5E-02	21	1.3E-02
**E2F**	TTTC/GGCGC	8	2.3E-02	66	2.0E-04
**NF-kB**	GGGGACTTTCCA	18	1.4E-01	218	6,90E-03
**N-Myc**	TCCCACGTGTCA/C/G	20	1.8E-03	198	4.0E-04
**MAX**	AAAA/CCACGTGGTTT	13	4.9E-02	149	1.2E-02
**USF**	GTCACGTGGC	51	1.3E-02	//	//
**Sp1**	A/GGGGGGCGGGGCC	68	2.8E-07	816	1.0E-10

a According to TELiS database (http://www.telis.ucla.edu/).

b AG: Number of genes analysed.

c Ranking according to the *p*-value (*p*<0.05) in incidence analyses.

//These transcription factors were not enriched in the promoters.

### Clustering and gene network analyses reveal peculiarities in the effect of DIG-MSK on ovarian carcinoma cells

**Figure 4:**
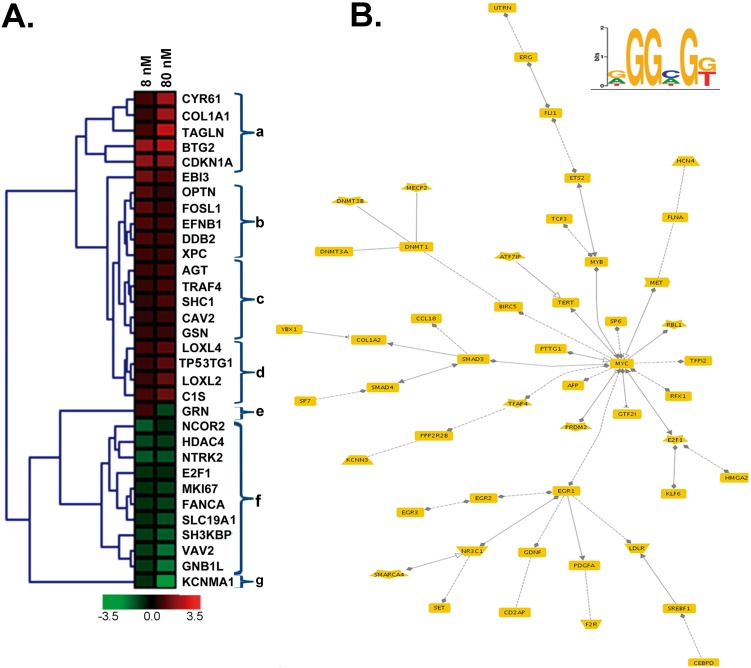
Effects of the treatment of A2780 cells with 8 nM or 80 nM DIG-MSK on a panel of genes characterized as being related to “ovarian neoplasm” and containing at least one putative Sp1-binding site in the proximal promoter region. (A) Hierarchical clustering of changes in gene expression for each treatment. Dendrograms show average-linkage hierarchical clustering based in Pearson correlation coefficients. For the sake of comparison, lowercase letters at the right side indicate “clusters” with shared characteristics that are detailed in Results. (B) Network generated by the Genomatix Pathway System (GePS) representing bibliographic relationships for Sp1 co-expressed gene profiles (*p* = 1.48E-03). Dashed lines indicate genes associated by co-citation, while continuous lines indicate genes associated by expert-curation. Filled diamonds and triangles indicate the promoter of gene “B” (the gene with the diamond/triangle) has a corresponding experimentally validated binding site for the transcription factor encoded by gene “A”. Open triangles indicate that the binding of a particular transcription factor to the gene promoter has not been described unambiguously. The sequence logo for the consensus Sp1 binding site, retrieved from JASPAR, is shown at the top right of panel B.

The effects of DIG-MSK on gene expression were also examined by clustering analysis of a selected set of genes. A hierarchical clustering, based on Pearson correlation coefficients, of 32 differentially expressed genes is shown in Fig. 4A. This set of genes was obtained after filtering the 278 up- and down-regulated genes influenced by both DIG-MSK concentrations (intersections in the Venn diagrams shown in Fig. 3A) using the Genomatix Pathway System (GePs). GePs identified 72 genes as belonging to “Ovarian Neoplasm” category within “Diseases/MeSH”. Ten of these genes were eliminated from the following analysis because their interaction with others was not evident. The remaining genes were then interrogated for the presence of putative Sp1 binding sites in their promoters using TELiS. Of them, 32 genes contained at least one Sp1-binding consensus sequence. Therefore, the clustered heat-map (Fig. 4A) summarizes the relationship between drug activity at each concentration and changes in the expression of Sp1-responsive genes relevant to ovarian cancer progression. Figure 4A offers a convenient way to visualize patterns of dissimilarity in the effects of either drug concentration. Dendrograms, showing average-linkage hierarchical clustering, clustered together several genes involved in common cellular pathways. For the sake of comparison, we have labeled those clusters using lowercase letters (Fig. 4A). Cluster labeled “a” contains genes up-regulated by either treatment, although 80 nM DIG-MSK had a superior enhancing effect. It encompassed genes related to various cell functions, as cell adhesion, migration, and proliferation, including genes involved in the control cell-cycle progression such as *CDKN1A* (*p21^WAF^*), which up-regulation was consistent with the transient halt of cells in G1 phase, described below. Most of these up-regulated genes, listed in Table 2, also contain putative Sp1-binding sites, although, in this case, Sp1 and DIG-MSK did not seem to compete directly for binding to consensus promoter sequences. Cluster “b” contains not only several genes that have been described to be highly expressed in ovarian cancer, but also *DDB2*, which enhanced expression would correlate with an augmented sensitivity of ovarian cancer cells to some chemotherapeutic agents [37]. Cluster “c” contains genes usually found highly expressed in ovarian carcinoma. DIG-MSK did not abrogate the expression of that particular group of genes. Cluster “d” contains genes whose expression can be induced in cellular stress conditions, as we may expect after drug treatment. We marked as “e” a single gene (*GRN*) that, although it clustered near others, it was peculiar in being up-regulated by 8 nM DIG-MSK and down-regulated by 80 nM DIG-MSK. *GRN* is a prognostic marker in epithelial ovarian carcinoma [38]. The large cluster “f” consists of genes that underwent a dose-dependent down-regulation. Among the genes clustered there was E2F1, a transcription factor, as well as genes that may contribute to tumorigenesis, including invasive ovarian carcinoma [39]. At the very edge of cluster “f”, but still closely related to it, *KCNMA1* was labeled as “g” (Fig. 4A). Amplification of this gene, which was strongly inhibited by DIG-MSK, has been associated with high cell proliferation and poor prognosis [40].

Genes down-regulated by 80 nM DIG-MSK were uploaded into GePS for transcriptional network analysis with sentence-level co-cited transcription factors annotation type (Genomatix Literature Mining). This provided insights into the functional connections among genes. Four transcription factors, Sp1, BIRC5, YY1 and BRCA1, showed significant (*p*<0.05) enrichment in that annotation type (Table S2). As a representative network, Fig. 4B presents genes regulated by Sp1, in which there was a dominance of *Myc* and *EGR1* as pivotal nodes.

Alongside the enrichment analysis in Gene Ontology terms described above (Tables 1 and 2), we undertook an unbiased enrichment comparison of the list of genes down-regulated by DIG-MSK with the list of terms available for A2780 human ovarian carcinoma cells in the Cancer Cell Encyclopedia [35]. We observed a strong and somewhat specific effect of DIG-MSK on genes that were up-regulated in A2780 cells, as deemed by the high statistical significance of the overrepresentation of DIG-MSK-abrogated genes among those up-regulated in A2780 cells (*p = *2.45E-14; Fisher’s exact test). DIG-MSK was rather effective in abrogating highly expressed genes in A2780 cells, which indicates its preference for interfering with over-expressed genes. This observation, a condition required to link its antitumor activity to changes in gene expression, was in keeping with the finding that other DNA-binding drugs show selective inhibiting effects on inducible genes [41], yet in our case the effect seemed related to a direct inhibition of transcription factor binding rather than due to downstream events.

### DIG-MSK challenges Sp1-binding to gene promoters

**Figure 5:**
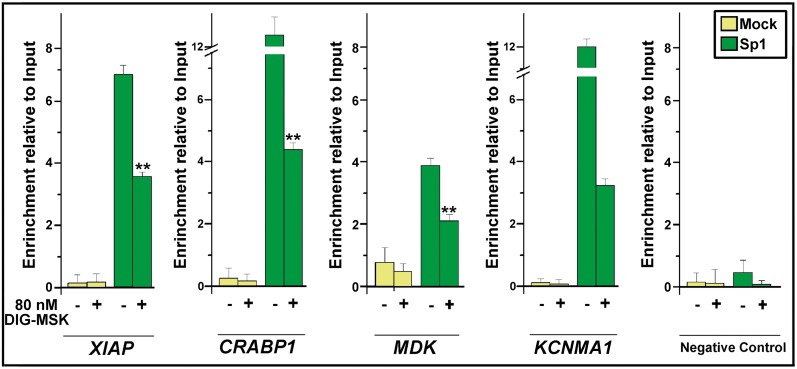
Chromatin immunoprecipitation analysis of Sp1 binding to the promoters of *XIAP*, *CRABP1*, *MDK* and *KCNMA1* genes in A2780 cells in the presence/absence of 80 nM DIG-MSK. ChIP was performed using an anti-Sp1 specific antibody. A DNA fragment that does not contain Sp1-binding sites was also immunoprecipitated as a negative control, as well as an unspecific immunoprecipitation using IgG (Mock). DNA in both the input and in the immunoprecipitated fractions was quantified by qRT-PCR. Data (mean ± SD from 3 independent experiments) are shown as the enrichment of input DNA in the immunoprecipitated fractions (**p<0.01; Student’s t-test).

Chromatin immunoprecipitation (ChIP) was used to measure Sp1 occupancy at the promoters of *XIAP*, *CRABP1, MDK* and *KCNMA1* genes, which were repressed by the treatments with 80 nM DIG-MSK. All of them are involved in the development of ovarian carcinoma [6]. A negative control was also included, which consisted of a down-stream region of the housekeeping *GAPDH* gene that lacks Sp1-binding sites. DIG-MSK decreased Sp1 binding to those promoters, with a superior effect on *KCNMA1* (Fig. 5).

### DIG-MSK changes the cell cycle traverse in A2780 cells and commits cells to dying

**Figure 6:**
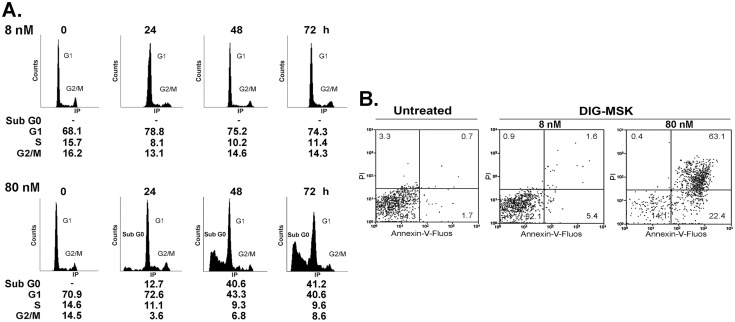
Cell cycle distribution and mechanisms of cell death. (A) Flow cytometry analysis of the time-dependent changes in cell cycle distribution of A2780 cells treated with either 8 nM or 80 nM DIG-MSK. Both adherent (attached) and detached (floating) cell populations were pooled together and their distribution in the different phases of the cell cycle quantified. (B) Analysis of cell death in A2780 human ovarian carcinoma cells treated with 8 nM or 80 nM DIG-MSK. This panel shows time-course bivariate flow cytometry analyses of control and DIG-MSK-treated cells stained with Annexin-V-Fluos and PI. Percentages of necrosis, apoptosis and secondary necrosis/apoptosis (double staining) are indicated inside the panels.

Changes in cell cycle distribution of A2780 cells after treatments with DIG-MSK were both concentration- and time-dependent (Fig. 6A). Adherent and detached cells were pooled together for cytometric analysis. A2780 cells treated with 8 nM DIG-MSK maintained a relatively uniform phase distribution throughout the experiment, with most cells in G1 phase, while 80 nM DIG-MSK produced a progressive increase in the sub-G0 population.

Flow cytometry bivariate-plots of Annexin-V-fluos and PI staining distinguished apoptosis from necrosis in A2780 cells treated with DIG-MSK for 72 h (Fig. 6B). Cells were mainly dying by secondary necrosis/apoptosis, which seemingly occurred after a fast apoptotic phase. Cell death was concentration-dependent with a higher effect of 80 nM DIG-MSK, which was also indicated by Trypan blue staining, and the increase in the G0-phase population (Fig. 6A).

### Changes in protein levels induced by DIG-MSK are consistent with changes in gene expression

**Figure 7:**
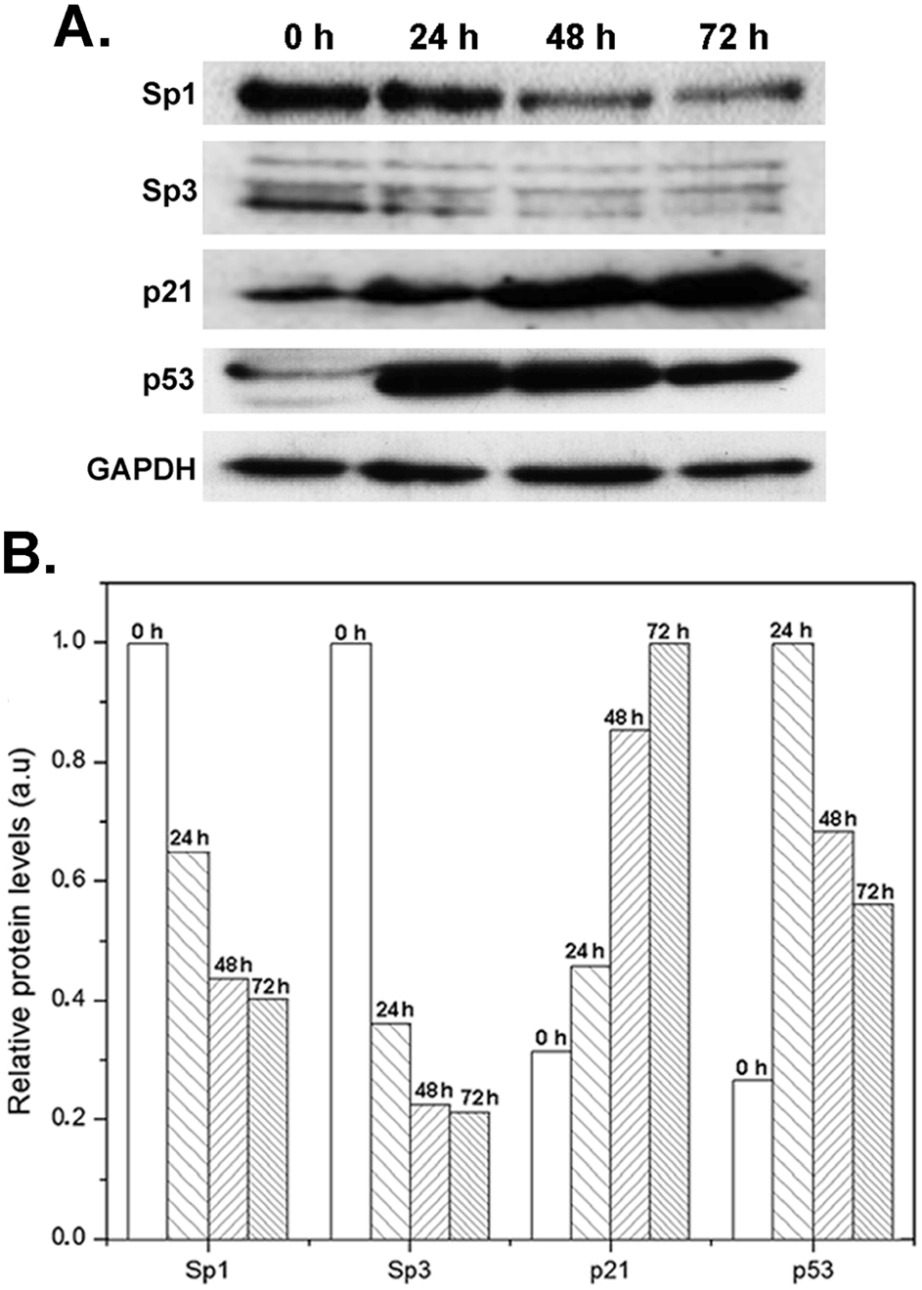
Immunoblotting analysis of protein levels. (A) Western blots showing changes in protein levels in A2780 cells treated with 80 nM DIG-MSK for the times indicated at the top of the panel. Experiments were performed in duplicate with similar results. (B) Quantification of the Western blots shown in panel A. It shows the time-dependent decrease in Sp1 and Sp3 (short isoform) protein levels, and the time-dependent enhancement of p53 and p21^WAF1^ (CDKN1A) protein levels.

Figure 7 presents some examples illustrating changes in protein levels after treatment of A2780 cells with DIG-MSK. Sp1 and Sp3 protein levels were in line with the down-regulation of gene expression by the drug. The levels of p53 increased after 24-h treatments, decreasing afterwards (Fig. 7B), consistent with cell arrest observed after 72 h (cf. Figs. 7A and 6A). Meanwhile, p21^WAF^ (*CDKN1A*) levels remained high. Altogether, changes in protein levels were fully consistent with both the array/qRT-PCR assays and the changes in the distribution of cells in the different phases of the cell cycle (Fig. 6A).

## Discussion

It is a matter of current interest whether small-molecule drugs targeting putative transcription-factor binding sites can cause sequential biological effects on gene expression, thus representing practical and reliable antitumor agents [5], [9]. Here, we have presented a genome-wide analysis probing the “anti-transcriptional” properties of the novel mithramycin analogue DIG-MSK, which had shown a promising *in vivo* antitumor profile [25].

We observed dose-dependent differences in the number of genes affected by DIG-MSK in A2780 cells. However, 105 genes were down-regulated regardless of the drug concentration (Fig. 3A, bottom panel). According to gene ontology (GO) analysis, they mainly belong to the categories of nucleic acid binding, transcription regulator activity and angiogenesis. Whereas 8 nM DIG-MSK had little effect on cell viability in up to 72-h treatments, the higher 80 nM DIG-MSK concentration, still in the nanomolar range, showed a remarkable time-dependent cytotoxicity, with most cells dying by apoptosis and secondary apoptosis/necrosis (Fig. 6B). At first glance, this would indicate that low concentrations of DIG-MSK are enough to produce specific effects on the expression of a relatively small number of genes, while higher concentrations affects a larger number of genes, which, in turn, augments cell death.

Transcription is the main target for DIG-MSK, as it also for other mithramycin analogues [10], [17], [29], [42]. The set of genes down-regulated by DIG-MSK comprises an extensive representation of genes that are up-regulated in untreated A2780 cells. Although the meaning of this observation has to be contemplated with caution, the genes overexpressed are a molecular signature of the transformed state of A2780 cells [35], thus they represent potential targets for therapeutic intervention in ovarian cancer [6].

The clustering analysis of a set of genes that displayed altered expression upon treatment (Fig. 4A) indicates that putative Sp1-binding sites, which are also potential binding sites for mithramycin analogues [10], [43], play a central role in both up-regulated and down-regulated genes. Among them, *GRN* (granulin epithelium precursor) is remarkable because it was up-regulated by 8 nM and down-regulated by 80 nM DIG-MSK. GRN has been characterized as a prognostic marker in ovarian cancer [38].

Functional network investigation of Sp1 co-expressed genes in DIG-MSK-treated cells highlights that *Myc* and *EGR1* are central nodes in the transcriptional network. They exhibited strong interactions with other genes, including *BIRC5*, *E2F1*, and members of the Sp-family genes (Fig. 4B and Table S2). All these genes encode transcription factors that are potential diagnostic biomarkers in ovarian cancer [7], [44]. Their activities were modulated by DIG-MSK (Table 3) as it also occurs in other cancer cell lines [29]. Moreover, Myc and EGR1 are involved in cancer development [45]. It is noteworthy that clustering gene expression (Fig. 4A) revealed that *KCNMA1* clustered with other genes down-regulated by any DIG-MSK concentration, but it was distinctly at the very edge of the rest of the cluster, denoting peculiarities of gene expression in presence of the drug; its expression was strongly inhibited by DIG-MSK. Such down-regulation is meaningful because *KCNMA1* high expression has been associated with cell proliferation and poor prognosis in cancer [40]. Hence, it is a candidate to be explored as target in ovarian cancer.

Here, we have shown that Sp1 is involved in the regulation of gene expression in A2780 human carcinoma cells treated with DIG-MSK. Differences in the down-regulating effects of 8 nM and 80 nM DIG-MSK can indicate differences in the binding affinity of Sp1 and the mithramycins for certain consensus C/G-rich tracts in DNA (i.e. DNA regions with high C+G content, in which these nucleotides are arranged in peculiar sequences), like in the *KCNMA1* promoter, which may result in variations in their concentration-dependent down-regulation (Fig. 4A). Given that Sp1 is overexpressed in cancer cells [46], its diminished levels upon treatments with DIG-MSK (Fig. 7) may result in tumor remission. In this regard, DIG-MSK activity resembles that of the structurally related MSK [17], [18], [29], [47]. DIG-MSK shows high activity in ovarian carcinoma cells [25] and Figs. 2 and 6B, while it has, in general, lower toxicity [25]. ChIP experiments show that the Sp1 occupies its binding sites in the promoters of *XIAP*, *CRABP1*, *MDK, and KCNMA1* (Fig. 5), and that DIG-MSK decreases Sp1 binding to them with a superior effect on *KCNMA1*, a gene associated with high proliferation in cancer cells [40]. The effect of DIG-MSK was also evident on the loading of Sp1 on the other gene promoters (Fig. 5): *XIAP* whose down-regulation can induce cell death [12], *CRABP1,* a gene involved in the development of ovarian carcinoma [11], and *MDK* that is a marker in ovarian cancer [14]. Besides, DIG-MSK modifies the interaction of other factors that also recognize C+G-rich regions in gene promoters. Some of them, including E2F1 and several members of the Sp-family of transcription factors, have been identified as biomarkers of ovarian cancer [44]. Among those transcription factors, E2F1 is member of one of the nodes of the cellular networks highly influenced by DIG-MSK (Fig. 4B).

Deregulated activity of transcription factors and the consequent overexpression of some genes frequently occur in cancer. In this context, MTA has been characterized a potent agent in abrogating the transcription driven by the EWS-FLI1 oncogenic transcription factor, a hallmark of the Ewing sarcoma family of tumors [48]. This is in keeping with the finding that binding to DNA of transcription factors other than the members of the Sp-family can be targeted by MTA and its analogues. Targeting over-expressed genes in ovarian cancer, including those of transcription factors such as Sp1 [7], [44] might be an appealing strategy to abrogate genes associated with resistance to other drugs [8]. As mentioned above, DIG-MSK shares with other mithramycin analogues the ability of shifting Sp1 binding, yet its better pharmacological profile [25] suggests that this new mithramycin analogue is a promising drug for the treatment of ovarian cancer.

## Supporting Information

Table S1 **Primers used for qRT-PCR.**
(PDF)

Table S2 **Networks affected by treatment with 8 nM and 80 nM DIG-MSK in human A2780 cells. Network analysis was performed using GePS.**
(PDF)
