# Supplementary material for: Genome-Wide Modulation of Gene Transcription in Ovarian Carcinoma Cells by a New Mithramycin Analogue
Source: PLoS One. 2014 Aug 11;9(8):e104687. doi: 10.1371/journal.pone.0104687 (PMC4128730; doi:10.1371/journal.pone.0104687)
Supplement: Table S1 — Primers used for qRT-PCR. (PDF) [file pone.0104687.s001.pdf]

Table S1. Primers used for qRT-PCR.

Gene	Primers (5' - 3')
SP1	for: CAGCTTCAGGCTGTTCCAAACT<br>rev: CTGCCAACTGACCTGTCCATT
SP3	for: GCGACAGGTGATTTGGCTTC<br>rev: CCATCGGTTTGGTGCTCCT
CDKN1A	for: TGTGATGCGCTAATGGCG<br>rev: CGAAGTTCCATCGCTCACG
TFDP1	for: CAACGAAGTGGCAGACGAGCTG<br>rev: GGTTGTCGGCAGCACTGAACTC
CCNA1	for: TCACCGTTCCTCCTTGGAAA<br>rev: TGAATGGTGAACGCAGGCT
TP53	for: CCCTTCCCAGAAAACCTACCA<br>rev: AAGAAGCCCAGACGGAAACC
MAPK1	for: GTTCTGCACCGTGACCTCAAGC<br>rev: ACAGGTGGTGTTGAGCAGCAGG
CCNB1	for: CAGGATAATTGTGTGCCCAAGA<br>rev: TGGCAGTGACACCAACCAGT
GTSE1	for: TCCCGAACAGCCTCCGTTG<br>rev: GGGCTCCAGGCAAAGGGAC
E2F1	for: AAGCGGCGCATCTATGACAT<br>rev: AATGAGCTGGATGCCCTCAA
BRCC3	for: CGTTACGGAAACATCGCTGTCG<br>rev: GGCCGCTGTGAAACAATGCTC
GAPDH	for: TCTGCCCCCTCTGCTGAT<br>rev: TTCTCATGGTTCACACCCATG
XIAP [a]	for: GAAAGAAGAAACACTGGAGC<br>rev: GCAGTGAGCACCTCGTAG
CREBP1 [a]	for: AGGGGCTGCGGGGGAG<br>rev: AGGAGAGCGCCCCCAAC
MDK [a]	for: GTTCCTGACCTCTGCCC<br>rev: GGAAGCCGGAGGGATCG
KCNMA1 [a]	for: GTGGAATCCAGTTGACAGC<br>rev: AGTGGGGAGGGGAGGAG

[a] Primers used for ChIP quantification.
