# Supplementary material for: Genome-Wide Modulation of Gene Transcription in Ovarian Carcinoma Cells by a New Mithramycin Analogue
Source: PLoS One. 2014 Aug 11;9(8):e104687. doi: 10.1371/journal.pone.0104687 (PMC4128730; doi:10.1371/journal.pone.0104687)
Supplement: Table S2 — Networks affected by treatment with 8 nM and 80 nM DIG-MSK in human A2780 cells. Network analysis was performed using GePS. (PDF) [file pone.0104687.s002.pdf]

Table S2. Networks affected by treatment with 8 nM and 80 nM DIG-MSK in human A2780 cells. Network analysis was performed using GePS.

Gene Set	Network[a]	*p*-value[b]	Associated Genes
**Genes up-regulated by both concentrations**	TP53	1.81E-07	*GADD45A; TP53INP1; CDKN1A; FAS; PCBP4; SPATA18; ZMAT3; DDB2; SESN1; GDF15; PLK3; S100A1; PLK2; TRAF4; TRIAP1; RRM2B; DRAM1; PCNA; BTG2; PRODH; DFNA5EDA2R; XPC; PTP4A1; DFNA5*
	DNA repair	4.14E-06	*GADD45A; TP53INP1; CDKN1A; DRAM1; DDB2; SESN1; GDF15; PLK3; PLK2; RRM2B; PCNA; BTG2; XPC; SAT1*
**Genes down-regulated by both concentrations**	Sp1	4.99E-03	*NFIX; TCF3; E2F6; E2F1; LRRFIP1; SP7; SLC19A1; HDAC4*
**Genes up and down-regulated by both concentrations**	Ovarian Neoplasm	1.47E-06	*GADD45A; CDKN1A; RHOC; S100A1; LIF; DDR1; TFIIA; FOSL1; SAA1; PLK2; TRAF4; NCOR2; OPTN; MTMR11; BTG2; GADD45B; EFNB1; INA; SAT1; CALD1; CYR61; EBI3; HLA- B; HDAC4; CORO2A; SLC19A1; GDF15; RPTOR; AHNAK; ANXA4; AGT; GNB1L; LOXL2; MAD1L1; SEMA3B; MKI67; KCNMA1; LRP1; NTRK2; CBX1; SHC1; LOXL4; C1S; E2F1; GRN; XPC; SH3KBP1; SYNE1; RCC1; CYP2E1; CAV2; TACC3; ACTA2; FAS; TAGLN; HBG1; AQP3; DDB2; HNRNPA2B1; PLK3; RXRA; SLC2A1; VAV2; CUX1; GSN; PCNA; HSP90AA1; TP53TG1; ADAMTSL4; COL1A1; FBXW7; FANCA*
**Genes down-regulated by 80 nM only**	BRCA1	2.37E-04	*IRF1; EGR1; SMARCA4; TRERF1; SREBF1; OAS2; E2F6; E2F1; PRKCZ; ADAMTS1; TAF4; POU2F1; HAS2; CENPW; FOXA1; REST; ZBTB7B; RFX1; TESC; TERT; UTRN; CREBBP; MECP2; TFAP4; MEF2D; SLC39A8; TFF1; LRRFIP1; NR2F1; DNMT3A; GDNF; COL1A2; PRDM2; POLD1; ATF7IP; CYP17A1; DDIT4; TFPI2; CD2AP; NFIX; NKX2-1; RNF4; GPC4; TIMP3; ETS2; GATA6; BACE1; NFYA; POU1F1; Sp5; ARHGEF10; SLC19A1; v-myc; Sp7; DNMT3B; PREX1; met; PCYT1A; ZBTB7A; PPP2R2; MIER1; HIF1A; ZBTB2; ZNF143; ZNF292; SALL2; HMGA2; CEBPD; HOXC4; v-ets; HDAC4; EPHX2; TCF3; DLC1; HCN4; TCF20; EGR2; EGR3; NFE2; HDAC7; NF1; AFP; Myc; Sp6*
	BIRC5	1.39E-04	*VEGFC; MYC; NOTCH1; CDKN2A; VEGFA; CASP7; ABCC5; CASP9; XIAP; DFFA; KDM5B; MCM5; RCC1; MKI67; BIN1; UPK2; USP10; BCL2A1; CCNG2; ERBB2; YY1AP1; HIF1A; MAGT1; E2F1; GPC3; FSCN1; JUP; TYMP; INCENP; XAF1; HMGB1; CENPF; MDK; CENPA; CFLAR; PDLIM7; TERT; COMP; FHIT; BCL2L11; CAV1; WTAP; PMAIP1; HSP90AA1; DIABLO; PML; DNMT3A; CDK6; BAGE*

Gene Set	Network[a]	*p*-value[b]	Associated Genes
	Sp1	1.43E-03	*MYC; SP7; DNMT3B; PREX1; MET; PCYT1A;ZBTB7Al; PARP1; PPP2R2B; MIER1; HIF1A; ZBTB2; ZNF143; ZNF292; SALL2; HMGA2; CEBPD; HOXC4; ERG; HDAC4; EPHX2; TCF3; DLC1; HCN4; TCF20; EGR2; EGR3; NFE2; HDAC7; NF1; SLC19A1; AFP; ARHGEF10; POLD1; ATF7IP; CYP17A1; DDIT4; TFPI2; CD2AP; NFIX; NKX2-1; RNF4; GPC4; TIMP3; ETS2; GATA6; BACE1; NFYA; POU1F1; SP5; IRF1; EGR1; SMARCA4; TRERF1; SREBF1; OAS2; E2F6; E2F1; PRKCZ; ADAMTS1; TAF4; POU2F1; HAS2; CENPW; FOXA1; REST; ZBTB7B; RFX1; TESC; TERT; UTRN; CREBBP; MECP2; TFAP4; MEF2D; SLC39A8; TFF1; LRRFIP1; NR2F1; DNMT3A; GDNF; COL1A2; PRDM2*
	YY1	1.32E-04	*SREBF1; MYC; PEG3; PIAS4; BACE1; COX7C; CXCR4; GON4L; POU2F1; CP; HOXA9; PARP1; CASP7; YY1AP1; BIN1; DLK1; E2F1; OCLN; ERBB2; SAP30; YAF2; NF1; CEBPD; HDAC4; MBTPS2; REST; DMD; CUX1; EGR1; HIST1H4A; FKBP1A; HOXB4; NFE2; CREBBP; MECP2; NFIL3; SLC1A3; GDNF; PRDM2; EGR2; GABPB1; PRRX1; MEIS1; RBPJ; CBX5; HIST1H1D*
**Genes up-regulated by 80 nM only**	TP53	3.03E-03	*PRODH; TPM3; CHMP4C; MPV17; CDKN2C; GLYAT; CDKN1A; IRF2BP2; KRAS; DPYD; C12orf5; PLK2; ERCC2; RPL27A; ZNF346; ERBB3; SLK; ATR; ING3; GAMT; RCHY1; LITAF; GDF15; RRM2B; ANXA6; ANXA5; POLH; EI24; TP53INP1; MCL1; NBN; MAP1LC3A; PSMD9; XPC; STMN1; DRAM1; LASP1; IDH1; PSMD10; APLP1; PPP1R13L; DCK; BCL6; BCL2; TERF2; NUPR1; OGG1; DDB2; SESN1; GADD45A; PTEN; ITGB4; FAS; PTP4A1; S100A1; GSTM1; S100A4; CCNG1; S100A6; ZMAT3; GSTT1; THBS1; TRAF4; CRYZ; PTPN13; BTG2; RNASEL; GLB1; NUAK1; ARL6IP1; UBE2N; BLCAP; DHCR24; UGCG; NT5C2; CYCS; FGF1; CD68; PLAGL1; ELF4; GML; PRKDC; ATM; GPNMB; FGFR3; MTBP; CDK1; PCBP4; PLK3; TOP2A; PRNP; PCNA; EDA2R; RAD17; HSP90AB1*
	DNA repair	7.96E-03	*RANBP9; TERF2; CDKN1A; BARD1SHMT1; TP53INP1; BMI1; CBX4; PLK3; NBN; RAD17; ALKBH1; ELF4; PLK2; PCNA; MAPKAPK2; ERCC2; PRKDC; ERCC4; POLH; BCL6; PPP1R15A; SAT1; LMNA; ATR; EP400; MAGED2; XPC; SMC3; OGG1; BTG2; CDC25B; DDB2; SESN1; RCBTB1; UBE2N; GADD45A; GLB1; MAPK9; ATM; RRM2B; TP53; GDF15; ZYX; TOP2A; RPAP3; SCARA3; CDK1; MPG; DRAM1*
	G1/S checkpoint	2.17E-03	*TGFB1; ATR; CDKN1A; TP53; ACTG1; ATM; HDAC1; CDK2; CDK4; CDK1*

[a]Based on Co-cited TFs, Co-cited genes, Signal transduction pathways and Diseases/Ovarian Neoplasm (MeSH).
[b]Fisher's exact test.
